# An Interesting Case Report on a Myocysticercosis Cyst

**DOI:** 10.7759/cureus.58884

**Published:** 2024-04-24

**Authors:** Ayushi Tayal, Sachin Daigavane, Nivesh Gupta

**Affiliations:** 1 Department of Ophthalmology, Jawaharlal Nehru Medical College, Datta Meghe Institute of Higher Education and Research, Wardha, IND

**Keywords:** taenia solium solitary lesion, parasitic disease, orbital cyst, extraocular muscle, orbital cysticercosis

## Abstract

One of the most dangerous parasite infections, cysticercosis, is found practically everywhere in the world. Cysticercus cellulosae is the larval stage of the swine tapeworm Taenia solium, which causes cysticercosis. Orbital or ocular cysticercosis (OOC) is an avoidable cause of blindness. There are two types of ocular cysticercosis: intraocular (in the anterior chamber, subretinal space, or vitreous) and extraocular (in the orbital tissues or subconjunctival space). Here, we report a rare case of extraocular muscle cysticercosis that presented as a solitary, well-defined lobulated mass near the medial canthus in the right eye and was well managed medically with antihelminthic drugs and corticosteroid therapy. The key to diagnosing myocysticercosis is orbital imaging. Although brain and ocular involvement in cysticercosis is common, extraocular muscle cysticercosis is extremely uncommon and mainly affects young people and children. Orbital pseudotumor, idiopathic myositis, and hydatid cysts are a few differential diagnoses for ocular cysticercosis. It is critical to recognize and treat such illnesses as early as feasible to avoid serious consequences. Public health measures are essential to eradicate this disease in the area.

## Introduction

One of the most dangerous parasite infections, cysticercosis is considered a neglected tropical disease and is found practically everywhere in the world [1,2]. In endemic locations, the reported rate of ocular involvement varies between 10% and 30% [3-6]. Neurocysticercosis (NCC) impacts the central nervous system, which frequently results in seizures and epilepsy [7]. The main cause of adult-onset epilepsy globally, in reality, is NCC [8]. Orbital or ocular cysticercosis (OOC) can also happen in addition to NCC, which is an avoidable cause of blindness [9]. 75-85% of cases of cysticercosis worldwide are related to OCC [10]. It is extremely uncommon for NCC and ocular cysticercosis to coexist [11]. There are two types of ocular cysticercosis: intraocular (in the anterior chamber, subretinal space, or vitreous) and extraocular (in the orbital tissues or subconjunctival space) [12,13].

## Case presentation

**Figure 1:**
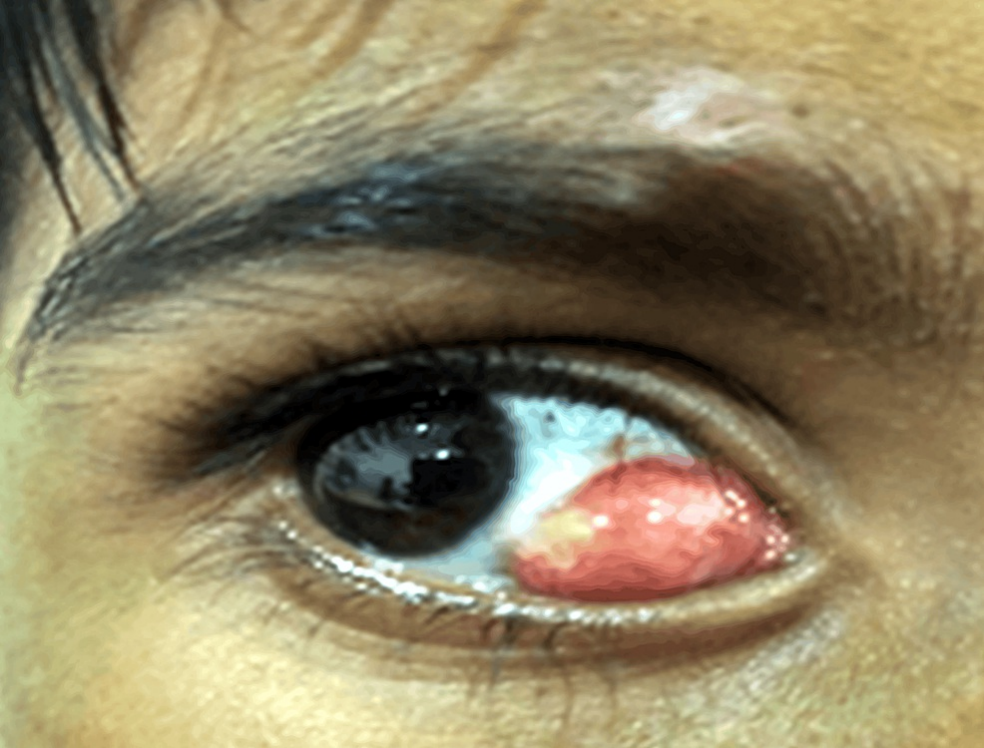
Ocular examination Image credit: Dr. Nivesh Gupta

A young eight-year-old boy, vegetarian by diet, reported to the out-patient department (OPD) of the District General Hospital, Gadchiroli, Maharashtra with major complaints of swelling in the right eye along with foreign body sensation for the last two months, which was insidious in onset, painless, and progressive. There was no history of trauma, diplopia, headache, malaise, fever, convulsions, or weight loss, and bending forward, coughing, or sneezing did not trigger the lump to get larger. The patient's vitals were stable, and he showed good orientation to time, place, and person during the routine physical examination. A solitary, pinkish-white, well-defined lobulated mass of approximately 10×6 mm in size near the medial canthus and 2 mm nasal to the limbus (on abduction) was discovered during a careful slit lamp examination of the right eye. Conjunctival congestion was observed nasal to limbus, spreading throughout the swelling. When palpated with a swab stick, it was non-tender, immobile, cyst-like, noncompressible, and nonreducible, with no pulsation or thrill. The cyst was producing ectropion on the medial side. The right eye's adduction was slightly decreased, but otherwise, the visual acuity was 6/6, N6 in both eyes with full and free extraocular muscle movements. The fundus viewed with complete mydriasis was normal. Rest ocular examination was normal. The left eye was within normal ranges. There were no swollen regional lymph nodes. A preliminary diagnosis of a parasitic cyst in the right eye was made. Figure 1 shows a solitary, well-defined lobulated mass of approximately 10×6 mm in size near the medial canthus and 2 mm nasal to the limbus in the right eye.

**Figure 2:**
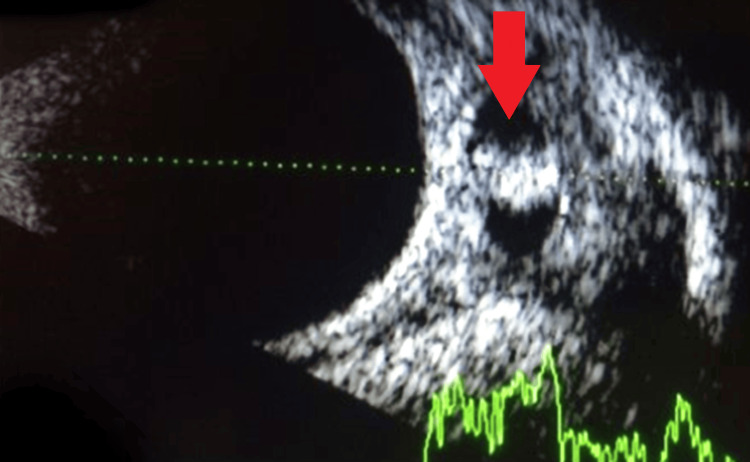
B-scan ultrasonography B-scan: brightness scan Image credit: Dr. Nivesh Gupta

**Figure 3:**
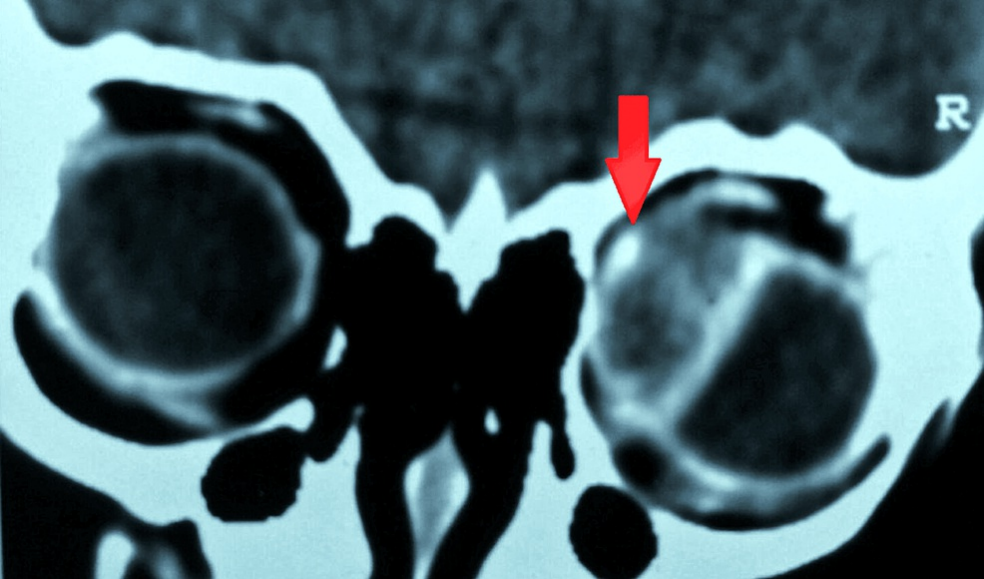
CT scan of orbit CT: computed tomography Image credit: Dr. Nivesh Gupta

Routine investigations on the case revealed an increase in ESR. The B (brightness)-scan ultrasonography revealed a hyper-reflective scolex in the medial rectus muscle, suggesting ocular cysticercosis, and a bulky medial rectus muscle with a well-defined anechoic cystic lesion bounded by a cyst wall. The computed tomography (CT) scan of the orbit showed a well-defined cyst involving the right-sided medial rectus suggestive of myocysticercosis. It also ruled out brain involvement and revealed no signs of NCC. The enzyme-linked immunosorbent test (ELISA) for anticysticercal antibodies in serum revealed a positive result. Cysts were also detected in the feces. There was no contribution from other laboratory studies. Figure 2 shows a B-scan image of a hyper-reflective scolex in the medial rectus muscle. Figure 3 shows a CT scan image of a well-defined cyst involving the medial rectus muscle (red arrow).

**Figure 4:**
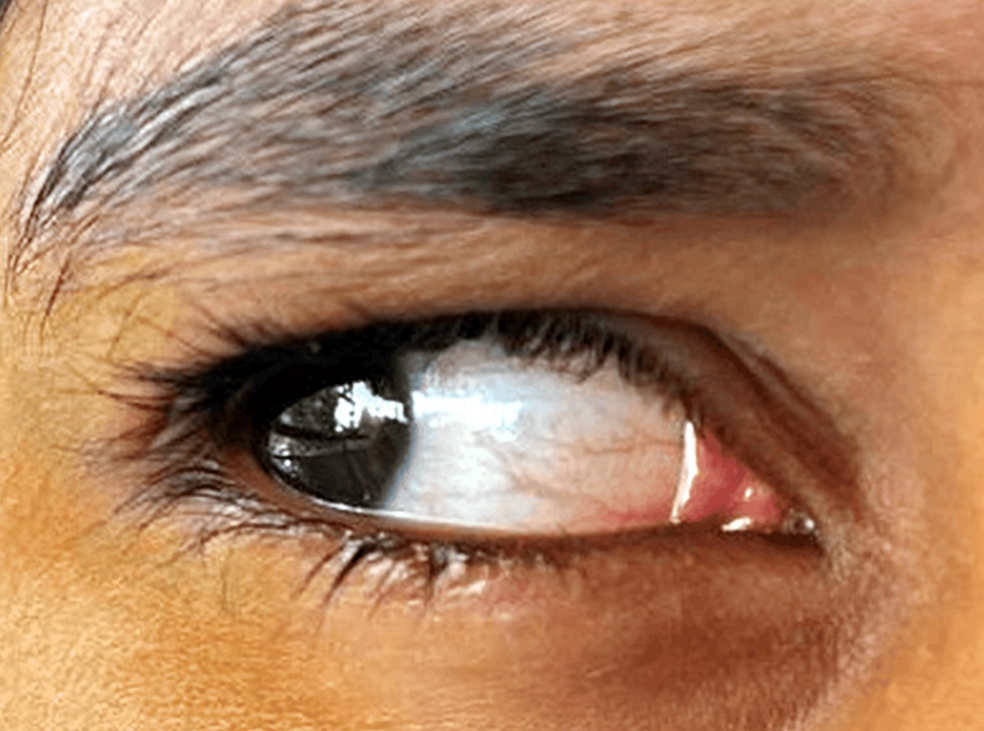
Follow-up ocular examination Image credit: Dr. Nivesh Gupta

A diagnosis of medial rectus ocular cysticercosis of the right eye was made. For four weeks, the patient was given oral prednisolone (1 mg/kg/day) and albendazole (15 mg/kg/day). After four weeks, oral prednisolone was gradually decreased over the course of the following month, and oral albendazole was discontinued. The patient was regularly followed up in OPD and substantial improvement in the size of swelling was observed two months after the above treatment was initiated. Figure 4 shows a follow-up image suggesting the disappearance of the cyst.

## Discussion

Cysticercosis is a prevalent issue in underdeveloped nations in Southeast Asia, South America, Africa, and Eastern Europe [14]. The native Indian population rarely becomes infected with cysticercosis, mainly because the non-vegetarian population uses pork sparingly and is primarily vegetarian. The lack of Indian reports of ocular cysticercosis is, therefore, not surprising. It exhibits a variety of clinical manifestations depending on the place of lodgment. The typical appearance of cysticercosis in the eyelid is a painless growing lump. Subconjunctival lesions typically manifest as hyperemic, occasionally fluctuating epibulbar masses. Cysticerci may also be seen in the anterior chamber attached to the iris or corneal endothelium or occasionally to the anterior lens capsule. Pupillary obstruction, which can result in glaucoma, could happen if the cyst is in the anterior chamber [15]. Cysticerci on the optic nerve head are quite rare. Although brain and ocular involvement in cysticercosis is common, extraocular muscle cysticercosis is extremely uncommon [16]. The primary cause of the extraocular cysticercosis clinical symptoms and signs is the inflammatory response to the larva and the space-occupying lesion it creates, which compresses the surrounding structures. Extraocular muscle cysticercosis typically manifests as persistent pain, redness, decreased ocular movement, diplopia (because of the misalignment of the visual axis), proptosis, and ptosis. Young people and children are frequently affected, with a median age of presentation at 13 years [17,18]. The medial rectus is the most often implicated extraocular muscle; however, the inferior rectus was the most frequently involved muscle in a different study [14,19].

The superior rectus muscles and levator palpebrae superioris muscle of the right eye were involved in an uncommon case of ocular cysticercosis, as reported by Agrawal S. and Somesh Ranjan et al. [20]. Verma R. and Jaiswal A. observed an odd correlation between ocular cysticercosis and numerous brain NCCs involving the superior rectus and levator palpebral superioris muscles [21]. Cysticercus cellulosae is the larval stage of the swine tapeworm Taenia solium, which causes cysticercosis. Humans can become infected with cysticercosis from the use of water or food tainted with Taenia solium eggs or rarely by autoinfection due to reverse peristalsis. Once ingested, the eggs hatch in the intestines, and the larvae can travel through the bloodstream to various organs, including the brain, skeletal muscles, eyes, and subcutaneous tissues [22]. Humans serve as accidental intermediate hosts in the life cycle of the parasite. Poor sanitation practices are a common cause of cysticercosis. Insensitive indicators include eosinophilia, inflammatory markers, and ELISA for antibodies specific to cysticercus.

The key to diagnosing myocysticercosis is orbital imaging. Finding high-amplitude spikes on A-scan ultrasonography that correlate to the cyst wall and scolex or an intracystic scolex on B-scan ultrasonography or a CT scan is diagnostic [14]. Because it was the most economical diagnostic modality, we decided to use ocular sonography (B-scan). The presence of a highly reflecting, core echodense, curved structure within the cyst indicating scolex narrows the differential diagnosis to cysticercosis as the etiological cause [23]. The ability of CT and ultrasound to identify scolices is similar [14]. Nevertheless, intracranial CT may be more advantageous since it can detect cerebral cysticercosis, which has an incidence of as much as 16.7% in a case series with myocysticercosis [24]. The initial medical therapy of intraocular cysticercosis with antihelminthic medicines such as albendazole or praziquantel is beneficial [25]. Sotelo et al. observed that albendazole was more effective than praziquantel while also being less expensive [26]. Clinical symptoms may worsen as a result of treatment-induced inflammation as the cyst involutes. Therefore, it is advised to administer corticosteroids concurrently to prevent an inflammatory reaction [27]. Despite medicinal therapy, a considerable minority of patients may still have functional limitations [28]. Therefore, the preferred treatment method is surgical removal of the parasite [29].

However, this is challenging due to the amorphous nature of the degenerating cyst, the cysts' attachment to the underlying orbital tissues, and the possibility of neurovascular injury [14]. Rarely affecting the eye, hydatid cysts are the differential diagnosis for ocular cysticercosis. The most common treatment for hydatid cysts in the eye is surgery since they are bigger and exhibit a "Spoke wheel pattern" and a two-layer cyst sign on ultrasonography. Orbital pseudotumor or idiopathic myositis is another crucial differential diagnosis, particularly when there is discomforting inflammation coupled with limited ocular movement. It is vital to distinguish orbital cysticercosis from other benign and malignant conditions that present as eye masses.

## Conclusions

In our situation, the cyst was located within the medial rectus muscle in a young patient, which is a very rare occurrence. From that perspective, the case under consideration is unique and uncommon. It is critical to recognize and treat such illnesses as early as feasible to avoid serious consequences. In this case, prompt diagnosis and treatment resulted in an early recovery. Public health measures, such as education, rigorous hygiene, and avoidance of food and water contaminated with human excrement, on a large scale are essential to eradicate this disease in the area.
